# Tissue-specific human beta-defensins (HBD)-1, HBD-2 and HBD-3 secretion profile from human amniochorionic membranes stimulated with Candida albicans in a two-compartment tissue culture system

**DOI:** 10.1186/1477-7827-10-70

**Published:** 2012-09-03

**Authors:** Veronica Zaga-Clavellina, Martha Ruiz, Pilar Flores-Espinosa, Rodrigo Vega-Sanchez, Arturo Flores-Pliego, Guadalupe Estrada-Gutierrez, Irma Sosa-Gonzalez, Iyari Morales-Méndez, Mauricio Osorio-Caballero

**Affiliations:** 1 Department of Cell Biology, Instituto Nacional de Perinatologia “Isidro Espinosa de los Reyes”, Mexico City, Mexico; 2 Department of Nutrition Research, Instituto Nacional de Perinatologia “Isidro Espinosa de los Reyes”, Mexico City, Mexico; 3 Department of Biochemistry and Molecular Biology, Instituto Nacional de Perinatologia “Isidro Espinosa de los Reyes”, Mexico City, Mexico; 4 Department of Infectology, Instituto Nacional de Perinatologia “Isidro Espinosa de los Reyes”, Mexico City, Mexico; 5 Department of Family Planning, Department of Gynecology and Obstetrics, Instituto Nacional de Perinatologia “Isidro Espinosa de los Reyes”, Mexico City, Mexico

## Abstract

**Background:**

During intrauterine infection, amniochorionic membranes represent a mechanical and immunological barrier against dissemination of infection. Human beta defensins (HBD)-1, HBD-2, and HBD-3 are key elements of innate immunity that represent the first line of defense against different pathogen microorganisms associated with preterm labor. The aim of this work was to characterize the individual contribution of the amnion (AMN) and choriodecidua (CHD) regions to the secretion of HBD-1, HBD-2 and HBD-3, after stimulation with *Candida albicans.*

**Methods:**

Full-thickness human amniochorionic membranes were obtained after delivery by elective cesarean section from women at 37-40 wk of gestation with no evidence of active labor. The membranes were cultured in a two-compartment experimental model in which the upper compartment is delimited by the amnion and the lower chamber by the choriodecidual membrane. One million of *Candida albicans* were added to either the AMN or the CHD face or to both and compartmentalized secretion profiles of HBD-1, HBD-2, and HBD-3 were quantified by ELISA. Tissue immunolocalization was performed to detect the presence of HBD-1, -2, -3 in tissue sections stimulated with Candida albicans.

**Results:**

HBD-1 secretion level by the CHD compartment increased 2.6 times (27.30 [20.9-38.25] pg/micrograms protein) when the stimulus with *Candida albicans* was applied only on this side of the membrane and 2.4 times (26.55 [19.4-42.5] pg/micrograms protein) when applied to both compartments simultaneously. HBD-1 in the amniotic compartment remained without significant changes. HBD-2 secretion level increased significantly in the CHD when the stimulus was applied only to this region (2.49 [1.49-2.95] pg/micrograms protein) and simultaneously to both compartments (2.14 [1.67- 2.91] pg/micrograms protein). When the stimulus was done in the amniotic compartment HBD-2 remained without significant changes in both compartments. HBD-3 remained without significant changes in both compartments regardless of the stimulation modality. Localization of immune-reactive forms of HBD-1, HBD-2, and HBD-3 was carried out by immunohistochemistry confirming the cellular origin of these peptides.

**Conclusion:**

Selective stimulation of amniochorionic membranes with *Candida albicans* resulted in tissue-specific secretion of HBD-1 and HBD-2, mainly in the CHD, which is the first region to become infected during an ascending infection.

## Background

During normal pregnancy, the human fetus must adapt to a potentially hostile environment in which both maternal and fetal tissues develop different and very complex adaptation strategies to avoid a potentially harmful response that can induce an alloimmune reaction between the mother and the fetus [1].

A growing body of clinical and experimental evidence supports the hypothesis that intrauterine infection during gestation is the principal causal factor in 25 to 40% of cases of preterm delivery; this condition jeopardizes the immunologic privilege of the fetus in the maternal-fetal interface, inducing an uncontrolled secretion of pro-inflammatory modulators that alter the immunologic/endocrinologic equilibrium of the maternal-placental unit [2,3].

The amniotic cavity is a space delimited by amniochorionic membranes, that represent a physical as well as a very active immunologic barrier. These capacities are required to guarantee the effective response against any immunological challenge, including an effective prevention and eventual contention of microorganisms that reach the uterine cavity through an ascending pathway from the cervical-vaginal region [4].

Amniochorionic membranes are a complex bi-laminated tissue constituted by an amniotic epithelium whose cells are in contact with the amniotic fluid and by a chorion zone rich in chorion laeve trophoblasts attached to the maternal decidual lining of the uterine wall; all these cellular populations are embedded in an extracellular matrix rich in collagen types I, III, IV, V, and VI, as well as nidogen and proteoglycans [5,6].

The amniochorionic membranes are minimally vascularized; hence their immunologic capacities have been attributed –in part- to the innate immune system [7]. There is experimental evidence suggesting that the innate immune system plays a key role in the defense mechanisms against any immune and infectious challenge in the fetal-placental interface. Different studies support that amniochorionic membranes have intrinsic antibacterial properties [8-10].

Natural antimicrobial peptides (AMPs) are effector molecules of the innate immune system that have anti-bacterial (gram negative and gram positive bacteria), anti-viral, and anti-fungal actions; generally, they are constituted by 12-50 amino acid residues, and most of them are positively charged and have amphipathic properties [11,12]. These properties are important for their microbial killing mechanism: the cationic character of AMPs results in electrostatic attraction to the negatively-charged phospholipids of microbial membranes and their hydrophobicity aids in integrating them into the microbial cell membrane, leading to membrane disruption [11,13].

Defensins are an evolutionarily ancient class of AMPs present in animals, plants, and fungi [14]. Mammalian defensins can be subdivided into three main classes according to their structural differences: alpha-defensin, beta-defensin, and theta-defensin. Human beta defensins (HBD)-1,-2, -3, and -4 represent the main group of antimicrobials expressed at mucosal surfaces by epithelial cells [15].

Different natural AMPs play an active role during an infection process and the amniochorionic membranes are especially active in their production. There is clinical and experimental evidence indicating that: 1) primary amnion epithelial cells express messenger RNA for HBD-1 to 3, as well as secretory leukocyte protease inhibitor (SLPI) and elafin. Treatment with IL-β –a pro-inflammatory cytokine associated with labor in normal and pathological conditions– increases significantly HBD-2 [16]; 2). Stimulation of FL cells (ATCC CCL-62), a human amnion-derived line with LPS induces up-regulation of mRNA of HBD-3 [17]; 3) human amniotic epithelial cells (HAEC), isolated from human fetal membranes with chorioamnionitis, secrete significantly more HBD-3 after stimulation with LPS than cells isolated from normal pregnancy membranes [18]; 4) HBD-2 concentrations are increased in the amniotic fluid of women with microbial invasion of the amniotic cavity (MIAC) [19]; 5) the concentrations of immunoreactive α-defensins, human neutrophil peptides (HNP)-1-3, bactericidal/permeability increasing protein (BPI), and calprotectin are significantly higher in patients with MIAC, preterm parturition, and preterm premature rupture of membranes (PPROM) [20]; 6) mRNA expression of alpha-defensin-1 and calgranulin B is significantly higher in fetal membranes of patients with preterm labor and histologic chorioamnionitis than in membranes of those without chorioamnionitis [21]; 7) elafin and HBD-3 increase in chorioamnionitis, and levels of HNP1-3 increase in plasma and amniotic fluid in women affected by microbial invasion of the uterus [22].

During pregnancy, HBD-1-3 plays a key role in the mechanisms that protect the maternal and fetal tissues and there is experimental evidence that they are expressed by the amnion’s epithelium, decidual, placental, and chorion trophoblasts from term pregnancies [10]. Evidence from our laboratory indicates that chorioamniotic membranes have tissue-specific capacities to synthesize and secrete HBDs. Using a two-compartment model of culture, we demonstrated that regardless of the primary site of stimulation with Streptococcus *agalactiae*, this bacterium induces secretion of HBD-2 and HBD-3 by both choriodecidual and amniotic regions; in contrast, HBD-1 remains without significant changes [23].

On the other hand, using the same experimental model, we demonstrated that *Gardnerella vaginalis* induces secretion of HBD-1 mainly in the amniotic compartment, and HBD-2 and HBD-3 were secreted by the choriodecidual region [24]. Stimulation with *Eshcerichia coli* elicits a similar secretion HBD-2 profile in both choriodecidual and amniotic regions, however, HBD-3 was secreted mainly by the choriodecidual region but only if the stimulus was applied on the amniotic side [25].

*Candida albicans* is considered a microorganism associated with bacterial vaginosis, a condition that has been identified as a previous condition for the development of intrauterine and/or intra-amniotic infections [4,26]. Chorioamnionitis cases due to yeast colonization remain rare despite 10-20% rate of vaginal yeast colonize during pregnancy. A growing body of clinical evidence indicates, however, that *C. albicans* can be related with chorioamnionitis [27-29] and neonatal infections [26,30,31].

Epidemiological data indicate that 69-94% of neonates with a birth weight less than 1500 g die because of *C. albicans*-induced infections. Moreover, there are reports of fetal death during midgestation (18-21 weeks) associated to sepsis by *C. albicans*[31,32].

HBDs exhibit anti-fungal activity against Candidas *spp*, including *C. albicans*[33-35], therefore, the rationale for the present study was to test the individual contribution of the amnion and the choriodecidual regions to the secretion of HBD-1, -2, and -3 after stimulation with *Candida albicans*. Our findings suggest that HBD-1-3 may provide protection against penetration by microorganisms into the immune-privileged amniotic cavity.

## Methods

### Biological samples

The Internal Review Board of the Instituto Nacional de Perinatolgía “Isidro Espinosa de los Reyes” (INPer IER) in Mexico City approved this study (#06151) and written informed consent was obtained from all participants.

Ten intact/whole term fetal membranes were collected from women who underwent elective cesarean section at term (37-39 weeks). All women were from an urban area of Mexico City, 23-34 years old, previously normotensive, with no history of diabetes mellitus, thyroid, liver, or chronic renal disease. They were cared for at the obstetrics out-patient service of the INPer IER. All women had uneventful pregnancies, with no evidence of active labor, cervical dilation or loss of the mucus plug. In addition, none had any clinical or microbiological signs of chorioamnionitis or of lower genital tract infection. Multi-fetal pregnancies were excluded from this study.

To confirm lack of infection, general microbiological analyses, including aerobic and anaerobic microorganisms, were conducted on the placenta and fetal membranes. Immediately after delivery, a sterile swab was rolled across randomly selected areas of the fetal membrane. The swabs were rolled onto Columbia agar with 5% sheep blood, which was used as a primary isolation medium for fastidious and non-fastidious aerobic microorganisms.

Appropriate selective media were included for the detection of specific pathogens, e.g. MacConkey II agar (*E. coli*). Gardnerella selective agar with 5% human blood (*G. vaginalis*), potato dextrose agar (*C. albicans*), agar with 5% human blood (group B streptococci), and chocolate II agar (*N. gonorrhoeae*) were included.

A CDC anaerobic 5% sheep blood agar plate was streaked to isolate obligate and facultative anaerobes, as well as microaerophilic bacteria, using a Gas Pak EZ anaerobic system. All culture media were purchased from BD (Germany) and were incubated following the manufacturer’s instruction. An additional swab was inoculated into Urea- Arginine LYO 2 broth (BioMérieux, Switzerland) to detect infection due to mycoplasma species.

Only membranes negative for aerobic and anaerobic microorganisms were used for this study.

### Amniochorionic membranes explants culture

This model has been previoously validated and published by our group [36] and reproduced by others [37,38]. Briefly, all specimens were collected under aseptic conditions in the operating room and transported to the laboratory within 10 min of delivery in sterile Dulbecco’s Modified Eagle Medium (DMEM) (Gibco BRL, Bethesda, MD) supplemented with 1X antibiotic-antimycotic solution (100 U/ml penicillin, 100 μg/ml streptomycin) (Gibco) and rinsed in sterile saline solution (0.9% NaCl**)** to remove adherent blood clots.

Segments representing all zones of the membranes were manually cut into 14-18 mm diameter discs and held together with silicone rubber and placed on the upper chamber of a Transwell device, which were placed in the 12-well culture plates (Costar, New York, NY).

One milliliter of DMEM supplemented with 10% fetal bovine serum (FBS), 1 mM sodium pyruvate, and 1X antibiotic-antimycotic solution (100 U/ml penicillin, 100 μg/ml streptomycin) (DMEM-FBS) (Gibco BRL) was added to each side of the chamber. The mounted explants were incubated in 5% CO_2_ at 37°C.

### Explants stimulations

The first 24 hours in culture were used to stablize the membranes after manipulation. On the second day the membranes in both compartments are washed with saline solution (0.9% NaCl) to remove FBS. After washing, the explants were cultured with 1 ml of DMEM/compartment suplemented with 0.2% of lactalbumin hydrolysate. Stimulation with the bacteria started immeditely upon addition of the culture medium.

Each experiment included the following set of chambers in triplicate: BASAL, control membranes in which 100 μl of saline solution (vehicle) was added to both culture medium in both compartments of the chamber; CHORIODECIDUA (CHD), 1 × 10^6^ CFU of *Candida albicans* was added only to the chorion compartment; AMNION (AMN), the yeast was added only to the amnion compartment; BOTH, the yeast stimulus was applied to both chorioamniotic and amniotic compartments simultaneously.

Co-incubation with *C. albicans* was done for 24 h, and the medium from both compartments of the chambers was collected and filtered (0.22 um); samples were aliquoted and stored at -70°C until assayed. All tissues were weighed and protein concentrations in all culture media were assessed with the Bradford method [39].

The inoculum size of 1 × 10^6^ CFU has been previoulsly standarized/published by our group to induce the secretion of a pro-inflammatory reaction, as well as the secretion of prostaglandin (PG)-E2 and matrix metalloprotease (MMP)-9 [40].

### Measurement of HBD1, HBD2 and HBD3 secretion

Concentrations of HBD1, HBD2, and HBD3 were determined by enzyme-linked immunosorbent sandwich assays (ELISA) kits (Pepro Tech, Rock Hill, NJ). Standard curves used were as follows: HBD-1, 15 to 1000 pg/ml with sensitivity of 4 pg/ml; HBD-2, 2 to 200 pg/ml with a detection limit of 1 pg/ml; and HBD-3, 10 to 2000 pg/ml with a sensitivity of 2 pg/ml.

Given that culture medium samples recovered from both compartments after stimulation did not contain fetal bovine serum and that these were filtered to remove any possible contamination with *C. albicans* and because HBD’s are secreted to the medium, the final concentration of each HBD was expressed per microgram of the total protein concentration of each sample.

### Immunohistochemistry

After stimulation with *Candida albicans*, the membranes were fixed, embedded in paraffin wax and 10-15 μm sections were processed for immunohistochemical staining using the following polyclonal antibodies; rabbit anti-hHBD-1 and anti-hHBD-3, and a goat anti-hHBD-2 (Pepro Tech). The antibodies were used at a 10ug/ml dilution. The same membranes without stimulation were used as controls.

Binding of primary antibodies was detected using the avidin-biotinylated peroxidase technique and biotinylated horse anti-rabbit IgG and horse anti-goat IgG antibodies (Vector, Burlingame, CA). Tissue sections were counterstained with Mayer’s hematoxylin and cover-slipped for evaluation by light microscopy.

### Statistical analyses

Descriptive statistics (mean, standard deviation, standard error, median, and range) were obtained for each variable. Data distribution was tested for normality using Kolmogorov-Smirnoff and Shapiro-Wilk tests. When distribution was normal, Student’s *t* test was used to analyze differences between treatments (i.e., control chorion vs stimulated chorion). Mann-Whitney’s U test was used when data were not normally distributed. In every case, a P value *≤* 0.05 was considered statistically significant. All statistical analyses were performed using SPSS 17 (IBM Corp., USA). Bars in the graphs represent median values and 25-75 interquartile range.

## Results

**Figure 1:**
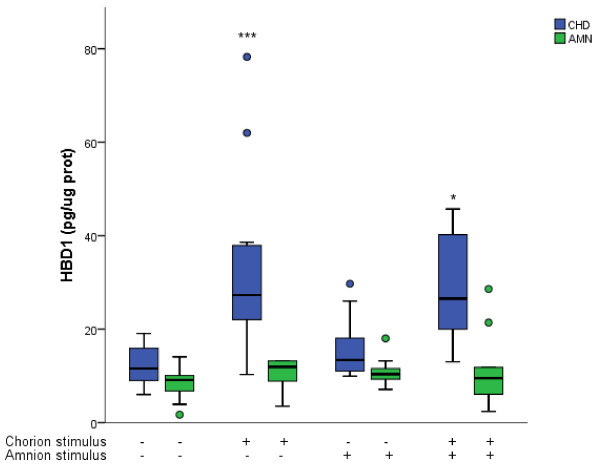
**Compartamentalized *in vitro *secretion of HBD-1 after selective stimulation with *Candida albicans.*** Antimicrobian peptide HBD-1 was measured in the medium from comparments delimited by the amnion (AMN) and the choriodecidua (CHD) under basal conditions and after the three different modalities of infection with 1 × 10^6^ CFU of *Candida albicans*. Boxes represent median concentration of HBD-1 with 25-75 interquartile range; outlier values are represented by ( · ); (*P ≤ 0.05, **P ≤ 0.01, ***P ≤ 0.001, n = 10). *Statistically different with respect to control value of choriodecidual compartment.

We evaluated HBD-1 secretion pattern in the culture medium after selective stimulation with *C. albicans* for 24 h. In comparison with basal secretion (11.57 [8.51-16.29] pg/μg protein) in the choriodecidual compartment, stimulation of the choriodecidual side of the membrane induced, at least, a 2.5-fold increase (27.3 [20.9-38.25] pg/μg) of HBD-1 in this compartment. A similar pattern was induced when the stimulus was applied to both AMN and CHD simultaneously (26.55 [19.4-42.5] pg/μg of protein) Figure 1.

Stimulation at the amniotic side of the membrane did not induce significant changes of HBD-1 secretion profile (P = 0.107) in either AMN (10.36 [9.06-12.12] pg/μg of protein) or CHD (13.40 [10.82-20.07]) regions. In comparison with control conditions, CHD was the most active in the secretion of HBD-1 and the amniotic side did not respond in a significant way under any of the three infection modalities Figure 1.

**Figure 2:**
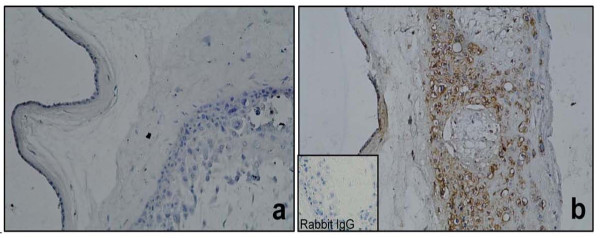
**Immunoreactivity of HBD-1 in human chorioamniotic membranes stimulated in both regions with *C. albicans*.** The first panel shows a non-stimulated control membrane (**a**. 20X). Panel **b** shows HBD-1 immunoreactive forms in membranes stimulated simultaneously with *C. albicans*.

Immunohistochemistry revealed an increase of immunoreactive forms of HBD-1 after simultaneous stimulation of amnion and choriodecidua regions with *C. albicans*. The strongest HBD-1 signal was localized in the trophoblasts of the chorion. Distribution of HBD-1 signal was similar in all stimulation conditions Figure 2.

**Figure 3:**
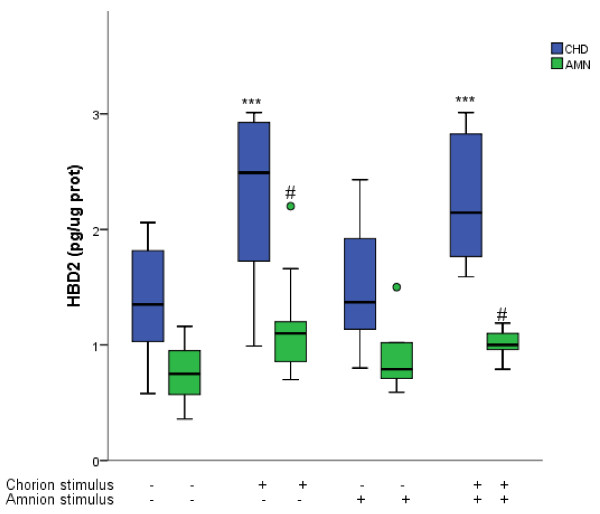
**Compartamentalized *in vitro *secretion of HBD-2 after selective stimulation with *Candida albicans*.** Antimicrobian peptide HBD-2 was measured in the medium from comparments delimited by the amnion (AMN) and the choriodecidua (CHD) under basal conditions and after the three different modalities of infection with 1 × 10^6^ CFU of *Candida albicans*. Boxes represent median concentration of HBD-2 with 25-75 interquartile range; outlier values are represented by ( · ) ; (*P ≤ 0.05, **P ≤ 0.01, ***P ≤ 0.001, n =10). *Statistically different with respect to control values of the choriodecidual compartment. #Statistically different with respect to control values of the amniotic compartment.

In comparison with basal levels, stimulation with *C. albicans* in the CHD region induced a significant increase of HBD-2 in both choriodecidual (2.49 [1.49-2.95] pg/μg of protein) and amniotic compartments (1.10 [0.85-1.20] pg/μg of protein). Yeast stimulation at the AMN side of the membrane did not induce any significant change (P = 0.25) in the pattern of HBD-2 secretion in either AMN or CHD Figure 3.

In comparison with basal levels in CHD (1.35 [0.99-1.92] pg/μg protein) and AMN (0.75 [ 0.53-0.96] pg/μg protein), simultaneous stimulation induced a significant increase of HBD-2 in chorionic and amniotic compartment (2.14 [1.67-2.91]) and (1.00-[0.9-1.13]) respectively Figure 3.

**Figure 4:**
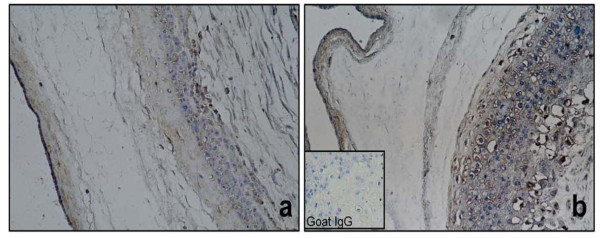
**Immunoreactivity of HBD-2 in human chorioamniotic membranes stimulated in both regions with *C. albicans*.** The first panel shows a non-stimulated control membrane (**a**. 20X). Panel **b** shows HBD-2 immunoreactive forms in membranes stimulated simultaneously with *C.albicans*.

Control tissues expressed immunoreactive forms of HBD-2 in the amniotic epithelium and trophoblast cells. Stimulation with *C. albicans* induced the increase of HBD-2 immunoreactive forms in both of these cells populations, especially within the choriodecidua region Figure 4.

**Figure 5:**
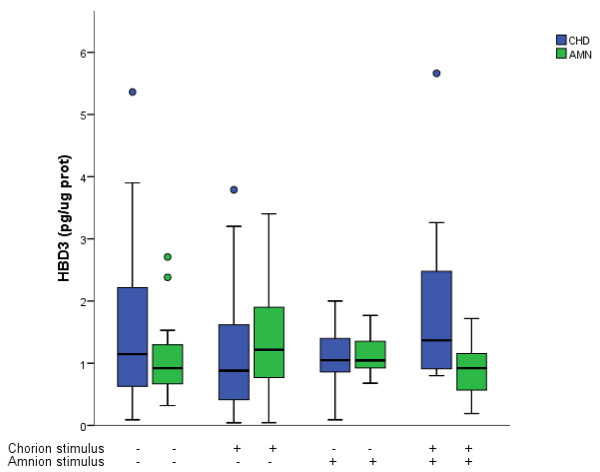
**Compartamentalized *in vitro *secretion of HBD-3 after selective stimulation with *Candida albicans*.** Antimicrobian peptide HBD-3 was measured in the medium from comparments delimited by the amnion (AMN) and the choriodecidua (CHD) under basal conditions and after the three differente modalities of infection with 1 × 10^6^ CFU of *Candida albicans* . Boxes represent median concentration of HBD-3with 25-75 interquartile range; outlier values are represented by ( · ) ; (*P ≤ 0.05, **P ≤ 0.01, ***P ≤ 0.001, n = 10).

**Figure 6:**
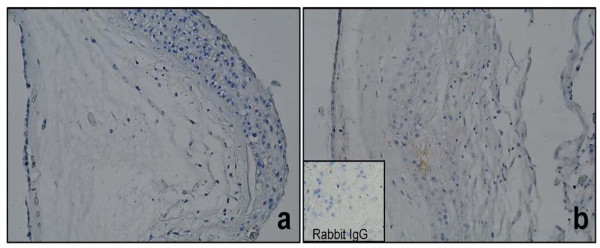
**Immunoreactivity of HBD-3 in human chorioamniotic membranes stimulated in both regions with *C. albicans*.** The first panel shows a non-stimulated control membrane (**a**. 20X). Panel **b** shows HBD-3 membranes stimulated simultaneously with *C.albicans*.

ELISA assays indicated that HBD-3 concentration in the membranes stimulated with *C. albicans* were not significantly different from those found in basal conditions regardless of stimulation conditions Figure 5. Immunolocalization of HBD-3 produced similar results Figure 6.

## Discussion

The immune innate system enables human amniochorionic membranes and other tissues of the fetal-maternal interface to recognize potentially infectious threats [41] and secrete AMP’s that act as the first defense line to delimit and control an infectious process [10,15]. The key role of fetal membranes as an effective barrier against *C. albicans* has been demonstrated in different animal experimental models [42,43].

*Candida albicans* has been identified as a microorganism isolated from the vagina, lower urinary tract, fetus-placental unit, and amniotic fluid during pregnancy and has been associated with an increased risk of premature rupture of membranes (PROM) and PPROM, which are associated with an increased risk of intra-amniotic infection; these are all conditions that negatively affect perinatal morbidity and mortality of newborns [44]. Infants with a birth weight of less than 1250 g comprise the group with the highest risk of developing pulmonary candidiasis [45].

In a study addressed to know whether *Candida* species can penetrate intact fetal membranes under *ex vivo* conditions, it was possible to demonstrate that *C. albicans* was the only one that, once inoculated into the maternal side, penetrated and passed to the fetal side, and caused degeneration of the structure of the membrane epithelium [46].

In the present study, we addressed the question of whether the amniochorionic membranes are able to respond to *Candida albicans* infection with the production of HBD-1, HBD-2, HBD-3, peptides that directly combat bacterial proliferation and survival. The experimental model used in this study allowed us to reproduce the different contact points between *Candida albicans,* ascending from the lower genital tract, and the uterine cavity; besides, we reproduced *in vivo* two compartments separated by a fully functional fetal membrane and analyzed the compartmentalized secretion in the compartment delimited by the amnion and that delimited by the choriodecidua [36].

The present work demonstrates that HBD-1 was the main defensin secreted by the membranes after stimulation with *C. albicans*. The ELISA and immunohistochemistry results support the hypothesis that the choriodecidua is the region more active in the synthesis and secretion of this defensin.

These results are supported by previous evidence indicating that mRNA and protein of HBD-1 are present in the amnion, decidua, and chorion trophoblast layers of amniochorionic membranes, being the trophoblast region the main source of endogenous antimicrobial molecules during pregnancy [10].

Wiechula et al. [47] demonstrated that the mRNA of HBD-1 is increased in the lavage of female genital tract collected from women with candidiasis, which is in agreement with studies done with human normal vaginal epithelia co-cultured with *C. albicans* that induce a significant secretion of HBD-1 and HBD-2 [48].

The abundant secretion of HBD-1 in our model could be supported by a possible relationship between pregnancy tissues and the genitourinary tract, which expresses high levels of these defensins [14].

There are reports indicating that under pathological conditions, such as chorioamnionitis and/or PROM, which have been associated with uncontrolled production of harmful pro-inflammatory cytokines, the pattern of HBD-1 synthesis and secretion, as well as its immuno-localization profile, does not change [10]. However in a recent work in our laboratory, using the same experimental model of two independent compartments, we demonstrated that stimulation with *G. vaginalis* induces upregulation of HBD-1 [24].

In contrast, a growing body of evidence indicates that HBD-2 is up-regulated by inflammatory cytokines in cells derived from reproductive tissues, including fetal membranes [25] and in other organs, such as skin [49], human endometrial epithelial cells [50], oral epithelial cells [51,52], respiratory epithelia [53], and different colonic epithelial cell lines [54].

In the present work, we confirmed that HBD-2 is present at basal level in the membranes, which agrees with previous evidence reported by King et al. [10]. On the other hand, stimulation with *C. albicans* induced the increase of secreted levels and immunoreactive forms of HBD-2 in both amniotic and choriodecidual compartments.

During pregnancy, HBD-2 is part of the immune machinery associated with the antimicrobial properties of the amniotic fluid; however, under pathological conditions such as MIAC, HBD-2 is up-regulated in the amniotic fluid regardless of the fetal membrane status (intact membranes or PROM) [19]. These results concur with evidence of our lab indicating that the infection of fetal membranes with *E. coli* –a microorganism associated with chorioamnionitis– elicited high secretion of HBD-2 by both amnion and choriodecidua regions [25].

Additional evidence indicates that transcription of the *HBD-2* gene is induced by IL-1β [54,55], a pro-inflammatory cytokine that is produced by the choriodecidual region of human fetal membranes in response to a selective stimulation with *C. albicans*[40]*.*

Additional experimental evidence indicate that HBD-2 elicits *in vitro* antifungal activity against *Candida albicans*, which is killed by a dose of 25 μg/ml of HBD-2 that is lethal for 90% of strains tested (LD_90_) [54,56] and in an energy-dependent and salt-sensitive manner without causing membrane disruption [57].

HBD-3 is a 4-kDa antimicrobial peptide originally isolated from human psoriatic lesion scales [12] whose strong expression has been demonstrated in keratinocytes and in tonsil tissue, whereas low HBD-3 expression was found in epithelia of the respiratory, gastrointestinal, and genitourinary tracts [58].

In the present study, we found that, independently from whether the yeast was added only to the amnion or to the choriodecidua or to both, stimulation with C*. albicans* did not induce any significant change in the HBD-3 secretion pattern, which was confirmed by immunohistochemistry.

It has been reported that HBD3 is up regulated by TNFα [59], however, previous evidence from our laboratory indicates that the stimulation of chorioamniotic membranes with *C. albicans* does not up-regulate the synthesis and secretion of TNFα [40].

Additionally, esophageal cell line OE21 stimulated with supernatants or directly co-cultured with *C. albicans* induces up-regulation of HBD-2 and HBD-3 expression, and the infection process involves divergent signaling events that differentially govern HBD-2 and HBD-3 [59]; these findings support our results indicating that the stimulation with *C. albicans* differentially up-regulates HBD-2 but not HBD-3, both defensins have been previously reported with strain-specific activity against *Candida sp.* including *C. albicans*[59,60].

Amniotic and choriodecidual regions are constituted by completely different cellular populations, which can explain –in part– the existence of tissue-specific HBD-1, HBD-2, and HBD-3 secretion patterns as part of the response to *Candida albicans*, this includes the basal protection of the three defensins and the up-regulation of HBD-1 and HBD-2 after stimulation with the yeast.

The present work and previous experimental evidence [23-25] support the concept that the human chorioamniotic membranes have immune capacities that include innate immune factors, such as HBDs, that are secreted in a tissue specific pattern as part of a complex mechanism of defense/protection against different pathogen microorganisms associated to preterm labor complicated with chorioamnionitis. The action of these molecules may limit the spread of pathogens between maternal and fetal compartments preventing the infection of the amniotic cavity.

Innate immune capacities in pregnancy tissues are key in the recognition, delimitation, and eventual destruction of pathogens that can damage the immunologic equilibrium at the maternal-placental interface. Differential capacities to recognize the pathogen are directly associated with receptors of the innate immune system, Toll like receptors (TLRs) are a family of pattern recognition receptors that respond to the presence of pathogen-derived products and are responsible in part for the control of antimicrobial expression [61,62].

There is evidence supporting the active role of the innate immune system, TLR-4 and TLR-2 are two key receptors in the recognition of linear structure of O-linked mannosyl residues [63] and β-(1,3)-mannosides, which are present in the acid-stable and acid-labile component of mannoproteins and phospholipomannans (PLM) [64] on the *Candida albicans* cell wall, respectively. Additionally fungal DNA is poorly methylated in contrast to mammalian DNA, and experimental evidence indicates that TLR-9 is involved in the recognition of *C. albicans*[62,65].

At the maternal-placental interface, TLRs play a key role as sentinels to detect different pathogens, these receptors are expressed not only in the immune cells but also in non-immune cells, including amniotic epithelium, villous and extra-villous trophoblasts, and decidual cells, and their expression pattern can vary according to the stage of pregnancy [61,62].

The expression and activity of TLR-2 and TLR-4 have been associated with spontaneous labor at term, as well as with preterm delivery with histological chorioamnionitis, indicating that these receptors play a key role in the innate immune mechanisms required to respond effectively against an immunologic-infectious process [61].

## Conclusions

Our results demonstrate that amniochorionic membranes respond differentially to *Candida albicans* infection. Amnion and choriodecidua secrete HBD-1 and HBD-2 as part of the innate immune defenses of this tissue.

The choriodecidua is the most responsive region to the infection, as it is the first tissue to be colonized by the microbial pathogen during an ascending intrauterine infection and it is the main barrier to progression of the infection into the amniotic cavity and eventually the fetus.

Tissue-specific capacities of these membranes to secret HBD-1 and HBD-2 represent the first line of defense in amniochorionic membranes and represent part of a very complex mechanism of antimicrobial protection that ensures an initial protection at times when infection may jeopardize the continuity of pregnancy.

## Abbreviations

AMN: Amnion; AMP: Natural antimicrobial peptides; BPI: Bactericidal permeability increasing protein; CHD: Choriodecidua; CFU: Colony forming unit; DMEM: Dulbecco modified eagle medium; ELISA: Enzyme-linked immunosorbent assay; FBS: Fetal bovine serum; HBD: Human beta defensins; HEC: Human amniotic epithelial cells; HNP: Human neutrophil peptide; LPS: Lipopolysacharide; MIAC: Microbial invasion of the amniotic cavity; PROM: Premature rupture of membranes; SLPI: Secretory leukocyte protease inhibitor; TLR: Toll like receptors.

## Competing interests

The authors declare that they have no competing interests.

## Authors’ contributions

VZC, MR, PFE, MOC, and AFP collected the samples, culture membranes, performed stimulation with the bacterium and ELISA assays. GEG and RVS performed the statistical analyses. ISG and IMM performed the microbiological control. VZC and MR participated in the design of the study, data collection, and analysis, as well as manuscript preparation. All authors have read and approved the final manuscript.
